# First Genome Sequence of Brucella abortus Biovar 3 Strain BAU21/S4023, Isolated from a Dairy Cow in Bangladesh

**DOI:** 10.1128/MRA.00446-19

**Published:** 2019-06-13

**Authors:** M. Sadequl Islam, Mohamed E. El Zowalaty, Arnoud H. M. van Vliet, Siddhartha Thakur, M. Minara Khatun, Sukumar Saha, M. Tanvir Rahman, Ayman Noreddin, M. Ariful Islam

**Affiliations:** a Department of Microbiology and Hygiene, Faculty of Veterinary Science, Bangladesh Agricultural University, Mymensingh, Bangladesh; b Infectious Diseases and Anti-Infective Therapy Research Group, Sharjah Medical Research Institute and College of Pharmacy, University of Sharjah, Sharjah, United Arab Emirates; c Department of Infectious Diseases, St. Jude Children’s Research Hospital, Memphis, Tennessee, USA; d Department of Pathology and Infectious Diseases, School of Veterinary Medicine, University of Surrey, Guildford, Surrey, United Kingdom; e Department of Population Health & Pathobiology, College of Veterinary Medicine, North Carolina State University, Raleigh, North Carolina, USA; f Comparative Medicine Institute, North Carolina State University, Raleigh, North Carolina, USA; University of Maryland School of Medicine

## Abstract

We report the genome sequence of Brucella abortus biovar 3 strain BAU21/S4023, isolated from a dairy cow that suffered an abortion in Savar, Dhaka, Bangladesh. The genome sequence length is 3,244,234 bp with a 57.2% GC content, 3,147 coding DNA sequences (CDSs), 51 tRNAs, 1 transfer messenger RNA (tmRNA), and 3 rRNA genes.

## ANNOUNCEMENT

Since its first description in 1906 (1), Brucella abortus remains one of the most important zoonotic and endemic diseases in several parts of the world (2). *Brucella* species are a group of aerobic, intracellular, small, non-spore-forming, nonencapsulated, and nonmotile Gram-negative coccobacilli (2, 3). They infect all livestock—avian, bovine, caprine, camelid, equine, ovine, and porcine (4, 5) and also wild animals (6, 7) and marine mammals (8). Human brucellosis causes a significant global public health and economic burden (9). Some species are subdivided into biovars; i.e., B. abortus species include eight biovars (1 to 7 and 9) (3). B. abortus causes infection predominantly in cattle, leading to substantial economic losses in dairy animals through stillbirths and decreased milk production (10). In Bangladesh, B. abortus infection is endemic in livestock and was reported to cause brucellosis in humans (11–13).

The genome sequence of B. abortus isolates from Bangladesh is essential because of its potential animal and public health impacts in this region. It allows in-depth analysis of genomic structure and will help us to understand its virulence, pathogenesis, host specificity, biotyping difference, and phylogenetic relationships and help to identify potential targets for the development of vaccines and diagnostics to prevent and control brucellosis.

Here, we report the first whole-genome sequence of B. abortus biovar 3 strain BAU21/S4023, isolated from a crossbred dairy cow (Bos taurus) in Bangladesh in March 2017. The *Brucella* strain was isolated from cow number 4023 (which suffered an abortion on a dairy farm in Savar, Bangladesh) by the streaking of a uterine discharge sample onto *Brucella* selective agar (HiMedia, India), which was then incubated at 37°C for 7 days in the presence of 5% CO_2_. Conventional bacteriological methods, classical biotyping, and enhanced AMOS-ERY PCR analysis confirmed the isolate as B. abortus biovar 3 (14, 15).

For genome sequencing, DNA was extracted from a single colony of strain BAU21/S4023 using a GeneJET genomic DNA purification kit (Thermo Fisher Scientific). DNA concentrations were quantitated using the Qubit 2.0 fluorometer for a double-stranded DNA high-sensitivity assay kit (Thermo Fisher Scientific, MA). Genomic libraries were constructed using a NEBNext Ultra DNA library prep kit (Illumina, Inc., San Diego, CA). The library size selection was 350 bp, and a paired-end (PE) sequencing strategy (2 × 150 bp) was performed by Apical Scientific (Selangor, Malaysia) using a HiSeq 4000 instrument (Illumina, Inc.). A total of 1,294 Mb (or ∼1.3 Gb) raw data reads were generated, and a total of 1.191 Mb (or ∼1.2 Gb) clean reads were obtained using Perl script to trim off Illumina adaptor sequences and remove low-quality reads. A total of 3.97 million reads passed the quality filter; reads averaged 150 bp in length and showed an average quality score above Q30 in more than 90% of the bases. Sequences were assembled using SPAdes version 3.11.0 (16) into 24 contigs at least 200 nucleotides (nt) long and a coverage of >10×, for a total of 3,244,234 bp with a GC content of 57.2%, an *N*_50_ value of 367,095, and an *L*_50_ value of 4 and containing 3,147 coding DNA sequences (CDSs), 51 tRNAs, 1 transfer messenger RNA (tmRNA), and 3 rRNA genes as identified by annotation using Prokka version 1.13 with default settings (17).

**FIG 1:**
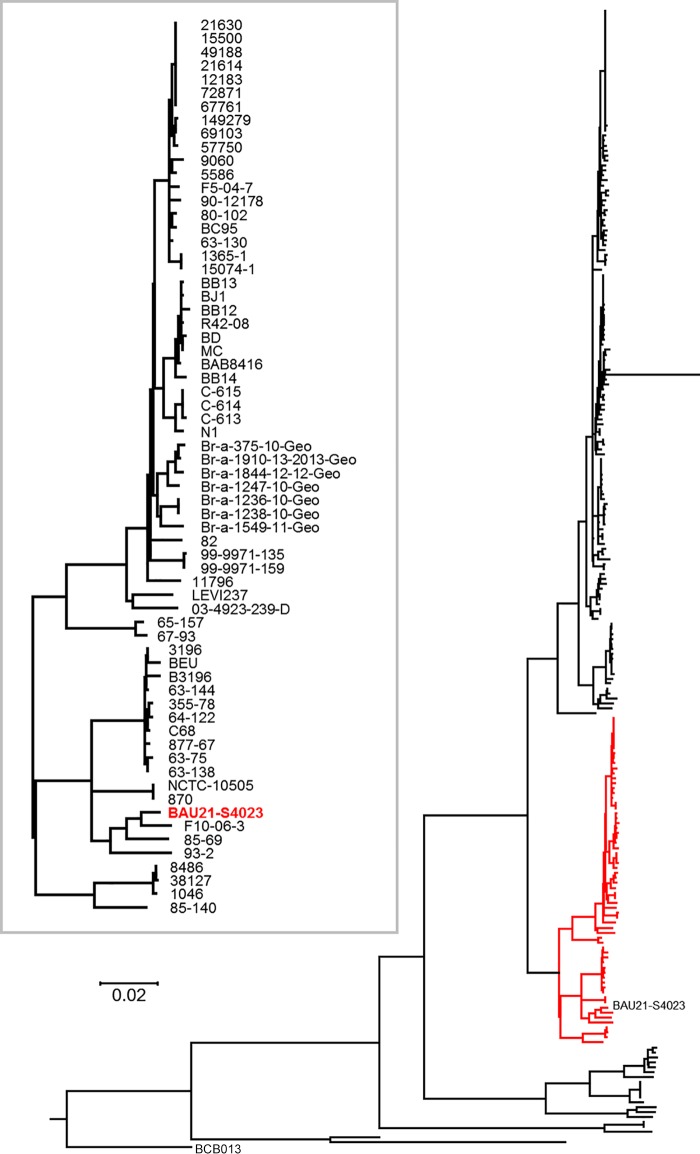
Phylogenetic tree of 229 B. abortus genome sequences based on core genome single nucleotide polymorphisms as identified using ParSNP. The position of the B. abortus BAU21/S4023 genome sequence is indicated in the tree, which was rooted with the genome sequence of B. abortus BCB013. The inset shows the part of the tree where the B. abortus BAU21/S4023 genome sequence clusters, with the closest relatives named.

A core genome single nucleotide polymorphism (SNP) tree of 228 genomes from GenBank was constructed to determine the relationship between the BAU21/S4023 strain and other available B. abortus isolates. B. abortus genomes were downloaded from the NCBI genome database using ncbi-genome-download version 0.2.9 (https://github.com/kblin/ncbi-genome-download), and core genome SNPs were identified and used for the construction of a phylogenetic tree using ParSNP version 1.2 (18) with the settings “-a 13” and “-x” as described previously (19). The genome of BAU21/S4023 was clustered closely with reference B. abortus genomes such as NCTC10505 (biovar 6), 870 (biovar 6), and C68 (biovar 9) (Fig. 1).

### Data availability.

This whole-genome shotgun project has been deposited at DDBJ/ENA/GenBank under the BioProject number PRJNA529883 and accession number SRJJ00000000. The version described in this paper is version SRJJ02000000. The sequences have been submitted to the Sequence Read Archive (SRA) under the accession number SRX5762378.
