# Correction for Islam et al., “First Genome Sequence of Brucella abortus Biovar 3 Strain BAU21/S4023, Isolated from a Dairy Cow in Bangladesh”

**DOI:** 10.1128/MRA.00779-19

**Published:** 2019-07-18

**Authors:** M. Sadequl Islam, Mohamed E. El Zowalaty, Arnoud H. M. van Vliet, Siddhartha Thakur, M. Minara Khatun, Sukumar Saha, M. Tanvir Rahman, Ayman Noreddin, M. Ariful Islam

**Affiliations:** a Department of Microbiology and Hygiene, Faculty of Veterinary Science, Bangladesh Agricultural University, Mymensingh, Bangladesh; b Infectious Diseases and Anti-Infective Therapy Research Group, Sharjah Medical Research Institute and College of Pharmacy, University of Sharjah, Sharjah, United Arab Emirates; c Department of Infectious Diseases, St. Jude Children’s Research Hospital, Memphis, Tennessee, USA; d Department of Pathology and Infectious Diseases, School of Veterinary Medicine, University of Surrey, Guildford, Surrey, United Kingdom; e Department of Population Health & Pathobiology, College of Veterinary Medicine, North Carolina State University, Raleigh, North Carolina, USA; f Comparative Medicine Institute, North Carolina State University, Raleigh, North Carolina, USA

## AUTHOR CORRECTION

Volume 8, no. 24, e00446-19, 2019, https://doi.org/10.1128/MRA.00446-19. Page 3: The following should be added to Acknowledgments. “M.E.E.Z., M.A.I., S.T., and A.H.M.V.V. contributed to writing the initial draft, M.A.I. submitted the strain for sequencing, and M.E.E.Z. analyzed the sequences and submitted the genome to NCBI GenBank and SRA.”

